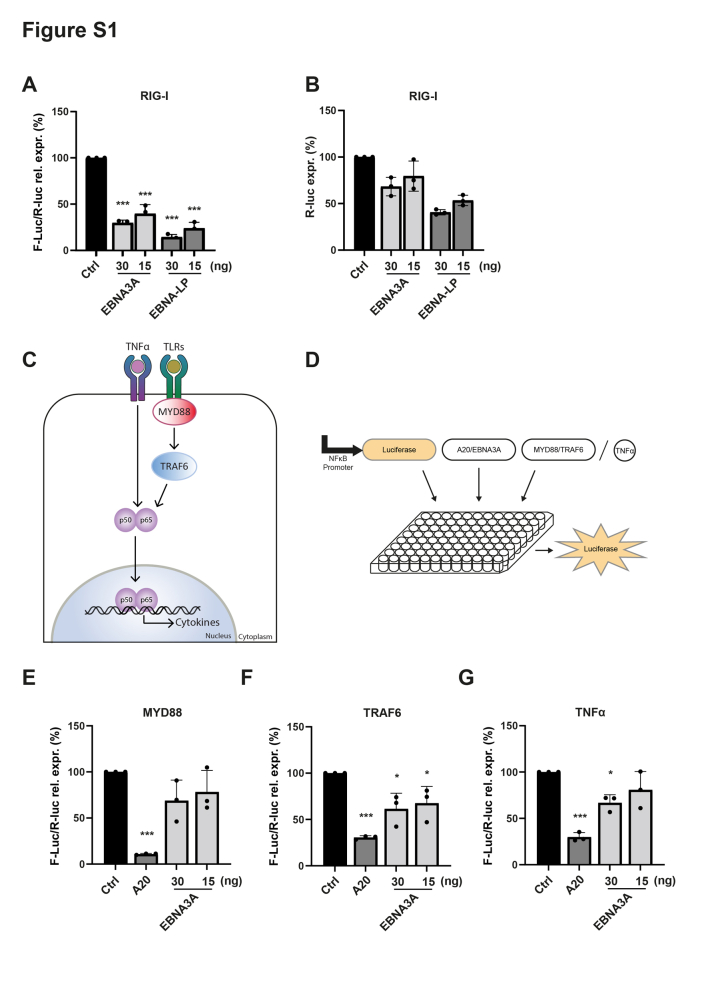# Epstein-Barr virus nuclear antigen EBNA3A modulates IRF3-dependent IFNβ expression

**DOI:** 10.1016/j.jbc.2024.107645

**Published:** 2024-08-08

**Authors:** Sanne L. Landman, Maaike E. Ressing, Anna M. Gram, Rayman T.N. Tjokrodirijo, Peter A. van Veelen, Jacques Neefjes, Rob C. Hoeben, Annemarthe G. van der Veen, Ilana Berlin

**Affiliations:** 1 Department of Cell and Chemical Biology, Leiden University Medical Center (LUMC), Leiden, the Netherlands; 2 Oncode Institute, Leiden University Medical Center (LUMC), Leiden, the Netherlands; 3 Center for Proteomics & Metabolomics, LUMC, Leiden, the Netherlands; 4 Department of Immunology, LUMC, Leiden, the Netherlands

**Keywords:** herpesvirus, Epstein-Barr virus, host-pathogen interaction, P300, IRF3, type I interferon, innate immunity, immune evasion, proximity labeling

## Abstract

Epstein-Barr virus (EBV), the causative agent of infectious mononucleosis, persistently infects over 90% of the human adult population and is associated with several human cancers. To establish life-long infection, EBV tampers with the induction of type I interferon (IFN I)-dependent antiviral immunity in the host. How various EBV genes help orchestrate this crucial strategy is incompletely defined. Here, we reveal a mechanism by which the EBV nuclear antigen 3A (EBNA3A) may inhibit IFNβ induction. Using proximity biotinylation we identify the histone acetyltransferase P300, a member of the IFNβ transcriptional complex, as a binding partner of EBNA3A. We further show that EBNA3A also interacts with the activated IFN-inducing transcription factor interferon regulatory factor 3 that collaborates with P300 in the nucleus. Both events are mediated by the N-terminal domain of EBNA3A. We propose that EBNA3A limits the binding of interferon regulatory factor 3 to the IFNβ promoter, thereby hampering downstream IFN I signaling. Collectively, our findings suggest a new mechanism of immune evasion by EBV, affected by its latency gene EBNA3A.

Various viruses have coexisted with humans throughout our species’ history. During this period of coevolution, viral pathogens have developed sophisticated strategies of host immune subversion. Due in part to these adaptations, viral infections continue to persist as major threats to global health. Among the highly prevalent viruses in the human population is the Herpesviridae family member, Epstein-Barr virus (EBV) (1). As part of its infection cycle, EBV establishes life-long persistence that involves two stages: latency and lytic reactivation. During latency, the virus resides quiescently in resting memory B cells, where it maintains the viral genome without engaging in virion production. Lytic reactivation in turn leads to the assembly and release of viral progeny (2). In adolescents, primary infection with EBV may initiate the disease onset of infectious mononucleosis (3). Individuals infected with EBV are at risk of developing lymphomas, carcinomas, and lymphoproliferative disease, possibly caused by the cell-transforming properties of the virus (4, 5). Infection with EBV is also associated with the development of multiple sclerosis (6, 7). Furthermore, the link between infection with SARS-CoV2 (the causative agent of COVID-19) and EBV reactivation is presently under investigation, as it may be associated with the symptoms experienced by patients suffering from long-term COVID-19 (8).

During persistent EBV infection, the presence of viral proteins and nucleic acids is sensed by pattern recognition receptors—cellular proteins that detect pathogen-associated molecular patterns. From the cytosol, (RIG-I)-like receptors (RLRs) and cyclic GMP-AMP synthase (cGAS) sense viral RNA and DNA, respectively. Because EBV’s genomic information is stored in the form of DNA, incoming viruses are at risk of being detected by cytosolic DNA sensors. Yet, (primary) human B-cells lack a functional cytosolic DNA sensing response (9). Instead, it has become evident that RIG-I can play a role in sensing EBV infection through the detection of small EBV-encoded RNAs and cellular 5S rRNA pseudogene transcripts, which are enriched and unmasked upon lytic reactivation of EBV (10, 11, 12, 13). Other herpesviruses have been reported to be sensed by RIG-I as well (14, 15). Upon RNA binding and oligomerization, RIG-I interacts with the mitochondrial antiviral signaling protein, which recruits the TANK-binding kinase 1 (TBK1) (16, 17, 18). Subsequently, phosphorylation of mitochondrial antiviral signaling protein by TBK1 stimulates recruitment of interferon regulatory factor 3 (IRF3) and instigates a succession of molecular events including phosphorylation, dimerization, and nuclear translocation of activated IRF3, which ultimately lead to the production and secretion of type I interferon (IFN) I (19). Secreted IFN I then binds to the interferon-α/β receptor (IFNAR) in an autocrine as well as paracrine manner, causing transcriptional upregulation of interferon-stimulated genes (ISGs) and induction of an antiviral cellular state (20). Proteins encoded by ISGs directly impair viral propagation, potentiate adaptive immune responses, and restrict the growth and proliferation of the host cell (21).

To counteract the antiviral effects of IFN I, viruses encode immune evasive genes that interfere with immune signaling at multiple levels. Within the γ-herpesvirus family, the Kaposi sarcoma-associated herpesvirus (KSHV) uses a cytoplasmic isoform of the latency-associated nuclear antigen to bind cGAS and inhibit downstream phosphorylation of TBK1 and IRF3 (22). Additionally, latent protein vIRF3 of KSHV inhibits IFNα production by interfering with the DNA binding activity of the transcription factor IRF7 (23). In the case of EBV, several lytic proteins have been shown to modulate innate immune signaling at the level of pattern recognition receptors, signaling intermediates, and transcription initiation (24, 25). During latency, EBV expresses a small number of genes, namely nuclear antigens EBV-nuclear antigen (EBNA)1, EBNA2, EBNA3A, EBNA3B, EBNA3C, EBNA-LP, membrane-associated proteins latent membrane protein (LMP)1 and LMP2, and several small RNAs. While expression of EBNA2 and the EBV-encoded RNAs leads to IFN I pathway activation (13, 26, 27), expression of LMP1 and LMP2 attenuates IFN I signaling by inducing degradation of RIG-I and the IFNAR, respectively (28, 29). The EBV-encoded microRNA BART6-3p also targets RIG-I, albeit at the transcriptional level, by targeting its mRNA for degradation (30, 31). Upon progression of latency, the number of viral gene products is reduced, which constitutes another way for EBV to evade immune activation (32). Collectively, these strategies intimate an underlying complexity and sophistication of herpesviruses in their avoidance of immune clearance, raising the possibility that other latency genes may also critically contribute to these efforts.

Here, we show that ectopic expression of EBNA3A inhibits IFN I induction downstream of RIG-I and TBK1. We find that EBNA3A binds to the interferon-β enhanceosome complex proteins CREB binding protein (CBP), P300 and IRF3 and prevents binding of IRF3 to its target DNA. Using mutational analysis, we demonstrate that the N-terminus of EBNA3A is essential for its inhibitory effect on the cellular IFN I response. Taken together, our findings shed new light on the latency arsenal available to EBV in its stride against antiviral immunity.

## Results

### EBV nuclear antigen EBNA3A inhibits the antiviral IFN I response

**Figure 1:**
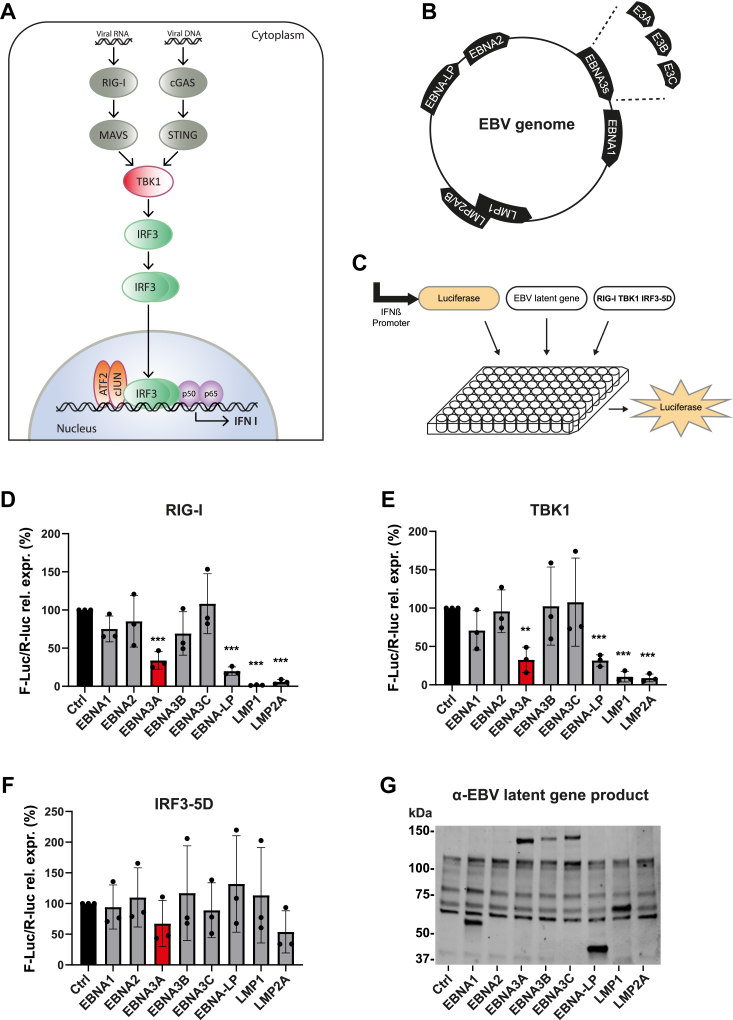
**Identification of EBV EBNA3A as an inhibitor of IFN I induction.***A*, schematic representation of cytosolic nucleotide signaling pathways leading to transcription of type I IFNs. *B*, overview of latent genes on EBV episome. *C–F*, overexpression screen for the effects of EBV latency gene products on IFN I induction. HEK293 cells were co-transfected with a reporter plasmid expressing the firefly luciferase gene under the control of the IFNβ promoter, a control plasmid expressing the renilla luciferase gene under the control of a constitutive promoter (for normalization purposes), and either (*D*) RIG-I, (*E*) TBK1, or (*F*) IRF3-5D, and the indicated EBV latency genes for 24 h. Firefly luciferase activity was measured and normalized to the renilla luciferase activity within each sample. Corrected firefly luciferase activity was then expressed relative to control set at 100%. n = 3 independent experiments, significance calculated using unpaired Student's *t* test. All graphs show mean ± SD. ∗∗*p* < 0.01; ∗∗∗*p* < 0.001. *G*, HEK293 cells were transfected for 24 h with the indicated His-tagged EBV latency genes. Cell lysates were analyzed by immunoblotting against His. Positions of marker standards are indicated. EBNA, EBV-nuclear antigen; EBV, Epstein-Barr virus; HEK, human embryonic kidney; IFN, type I interferon; IRF3, interferon regulatory factor 3; RIG-I, retinoic acid-inducible gene I; TBK1, TANK-binding kinase 1.

To determine whether EBV encodes latency-associated genes that prevent activation of the IFN I response, we performed a screen in human embryonic kidney (HEK293) cells equipped with a reporter construct encoding the Firefly luciferase under the control of the IFNβ promoter as a proxy for IFN I induction (Fig. 1, *A–C*). Ectopic expression of RIG-I, TBK1, or a constitutively active mutant of IRF3 (IRF3-5D)—in which serine or threonine residues at positions 396, 398, 402, 404, and 405 were replaced by phosphomimetic aspartic acid residues (33) – in combination with latent EBV genes in the reporter cells was used to assay IFN I induction (Fig. 1, *D–F*). In agreement with previous reports, overexpression of LMP1 and LMP2A strongly decreased RIG-I- and TBK1-dependent IFN I induction in our reporter assay (Fig. 1, *D* and *E*) (28, 29). Also, EBNA3A (red bars) and EBNA-LP showed robust inhibition of IFN I induction in the presence of either RIG-I or TBK1 (Fig. 1, *D* and *E*), positioning these EBV gene products as additional candidate viral inhibitors of the IFN I pathway. Interestingly, none of the viral genes tested significantly inhibited IFN I induction by IRF3-5D (Fig. 1*F*), implying that interference occurs upstream of IRF3 activation, or that mutations in IRF3-5D render it resistant to viral interference. To ensure that lack of IFN I inhibition by EBNA1, 2, 3B, and 3C was not due to insufficient ectopic expression of the indicated constructs, we assayed protein abundance of all latent genes tested by immunoblotting (Fig. 1*G*). Robust expression was observed for all, except EBNA2, which was therefore excluded from further analysis.

Next, we titrated increasing amounts of EBNA3A and EBNA-LP into our reporter assay. Both EBNA3A and EBNA-LP perturbed IFNβ promoter activity in a dose-dependent manner (Fig. S1*A*). However, expression of low amounts of EBNA-LP (15 ng) already reduced Renilla luciferase activity (transfection control) by 50% (Fig. S1*B*), pointing to cytotoxicity. To avoid confounding factors, we chose to focus on EBNA3A as a novel inhibitor of IFN I signaling. We also examined the effect of EBNA3A on NFκB signaling using the NFκB-responsive Firefly luciferase reporter plasmid and expression of a known inhibitor of NFκB pathway activation, the deubiquitinase A20, as a positive control (Fig. S1, *C* and *D*) (34). EBNA3A reduced activation of NFκB signaling in HEK293 cells (either ectopically expressing MYD88 and TRAF6 or stimulated with TNFα) to a lesser extent than in the case of IFN I induction (Fig. S1, *E–G*). These data suggest that EBNA3A constitutes a potent inhibitor of IFN I induction, while only moderately affecting NFκB signaling.

### EBNA3A inhibits IFN I signaling upstream of the IFNAR

**Figure 2:**
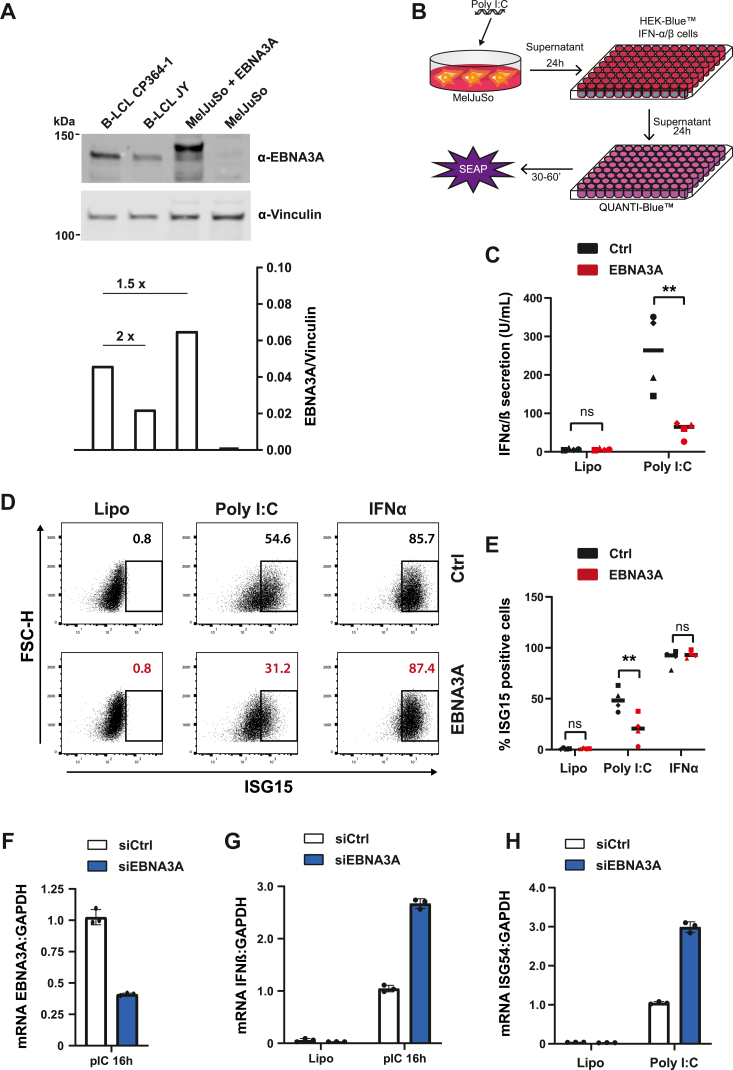
**EBNA3A inhibits IFN I signaling upstream of the IFNAR.***A*, MelJuSo cells stably transduced with EBNA3A exhibit comparable levels of EBNA3A to B-LCLs. An equal number of MelJuSo and B-LCL cells were lysed and subjected to SDS-PAGE analysis and immunoblotting. Protein levels of EBNA3A were quantified and normalized to the Vinculin signal. Positions of marker standards are indicated. *B* and *C*, effect of EBNA3A expression on IFN I activity. MelJuSo cells stably expressing EBNA3A or GFP were lipofectamine transfected for 24 h with Poly(I:C) or treated with lipofectamine alone (Lipo). IFN I secretion was measured by an IFN I bioassay using HEK-Blue IFN-α/β reporter cells and plotted as secreted units per mL of supernatant. n = 4 independent experiments, significance calculated using paired Student's *t* test. Graphs show mean ± SD. *D* and *E*, effect of EBNA3A expression on ISG15 upregulation. EBNA3A-expressing or control MelJuSo cells were lipofectamine transfected with Poly(I:C) *versus* treated with lipofectamine alone (Lipo) or stimulated with IFNα for 24 h. Intracellular ISG15 protein expression was measured by flow cytometry (*left panel*) and reported as % of ISG15 positive cells. n = 4 independent experiments, significance calculated using paired Student's *t* test. Graphs show mean ± SD. *F–H*, increased induction of IFNβ and ISG54 transcripts in response to EBNA3A depletion. MelJuSo cells were transfected with a siCtrl or siEBNA3A for 72 h. 16 h prior to collection, cells were transfected with Poly(I:C) or treated with lipofectamine only (Lipo). RT-qPCR analysis was used to confirm silencing of EBNA3A (*F*) and to monitor the type I IFN response by IFNβ (*G*) and ISG54 (*H*) transcripts, all normalized to a housekeeping gene (GAPDH). Data are means ± SD from a representative of three biological replicate experiments. ∗∗*p* < 0.01. EBNA, EBV-nuclear antigen; HEK, human embryonic kidney; IFN, type I interferon; IFNAR, interferon-α/β receptor; ISG, interferon-stimulated gene.

To explore the physiological relevance of EBNA3A in the context of IFN I induction, we proceeded to examine latently infected B-lymphoblastoid cell lines (B-LCLs), JY, and CP364-1 (9, 35). Both cell lines readily express EBNA3A (Fig. 2*A*), and an EBNA3A deficient cell line has been reported in the latter background to facilitate loss of function studies (35). Direct engagement of the IFNAR through the addition of recombinant IFNα to the culture medium resulted in robust ISG15 responses in both JY and CP364-1 cells (Fig. S2*A*). We next tested the ability of both B-LCL lines to induce upregulation of ISG15 (an exemplary ISG) downstream of the RIG-I signaling cascade. To this end, poly(I:C), a synthetic analog of double stranded RNA, was introduced into the cytoplasm of B-LCLs. In JY cells, this led to an increased ISG15 signal, while CP364-1 cells remained unresponsive to poly(I:C) stimulation (Fig. S2*B*). Due to their lack of responsiveness to poly(I:C) stimulation, CP364-1 could not be further investigated in the context of the IFN I pathway. Instead, we aimed to assay the effects of EBNA3A depletion in JY cells. However, in line with other reports on the resistance of lymphocytes towards lipid-mediated transfection as well as viral transduction (36, 37), we were unable to downregulate EBNA3A in JY cells (Fig. S2*C*).

Based on the technical challenges described earlier, we constructed a MelJuSo model cell line, wherein EBNA3A was stable and expressed at levels comparable to those of CP364-1 and JY cells (Fig. 2*A*). MelJuSo human melanoma cells have been previously investigated in the context of innate immune signaling (38) and are highly amenable to RNA-mediated interference (39), and thus constitute a suitable alternative. First, to investigate whether EBNA3A can modulate secretion of IFN I and consequent upregulation of ISG15, we introduced poly(I:C) into mock-transduced MelJuSo cells *versus* those transduced with EBNA3A and measured secretion of IFN I using a bioassay (Fig. 2*B*). As expected, cells expressing EBNA3A produced significantly less IFN I (Fig. 2*C*). Similarly, intracellular protein levels of ISG15 were reduced in cells expressing EBNA3A in response to treatment with poly(I:C) (Fig. 2, *D* and *E*). By contrast, stimulation with IFNα was not significantly affected by EBNA3A (Fig. 2, *D* and *E*), indicating that inhibition of IFN I signaling by EBNA3A occurs upstream of the IFNAR. To complement these data, we assayed the effects of siRNA-mediated depletion of stably expressed EBNA3A in MelJuSo cells. Silencing EBNA3A dramatically improved poly(I:C)-dependent induction of IFN I and ISG54 transcripts (Figs. 2, *F*–*H* and S2*D*), implying that the presence of EBNA3A represses the RIG-I-mediated transcriptional program. Taken together, gain- and loss-of-function experiments position EBNA3A as a negative regulator of IFN I pathway activation.

### EBNA3A interacts with P300

**Figure 3:**
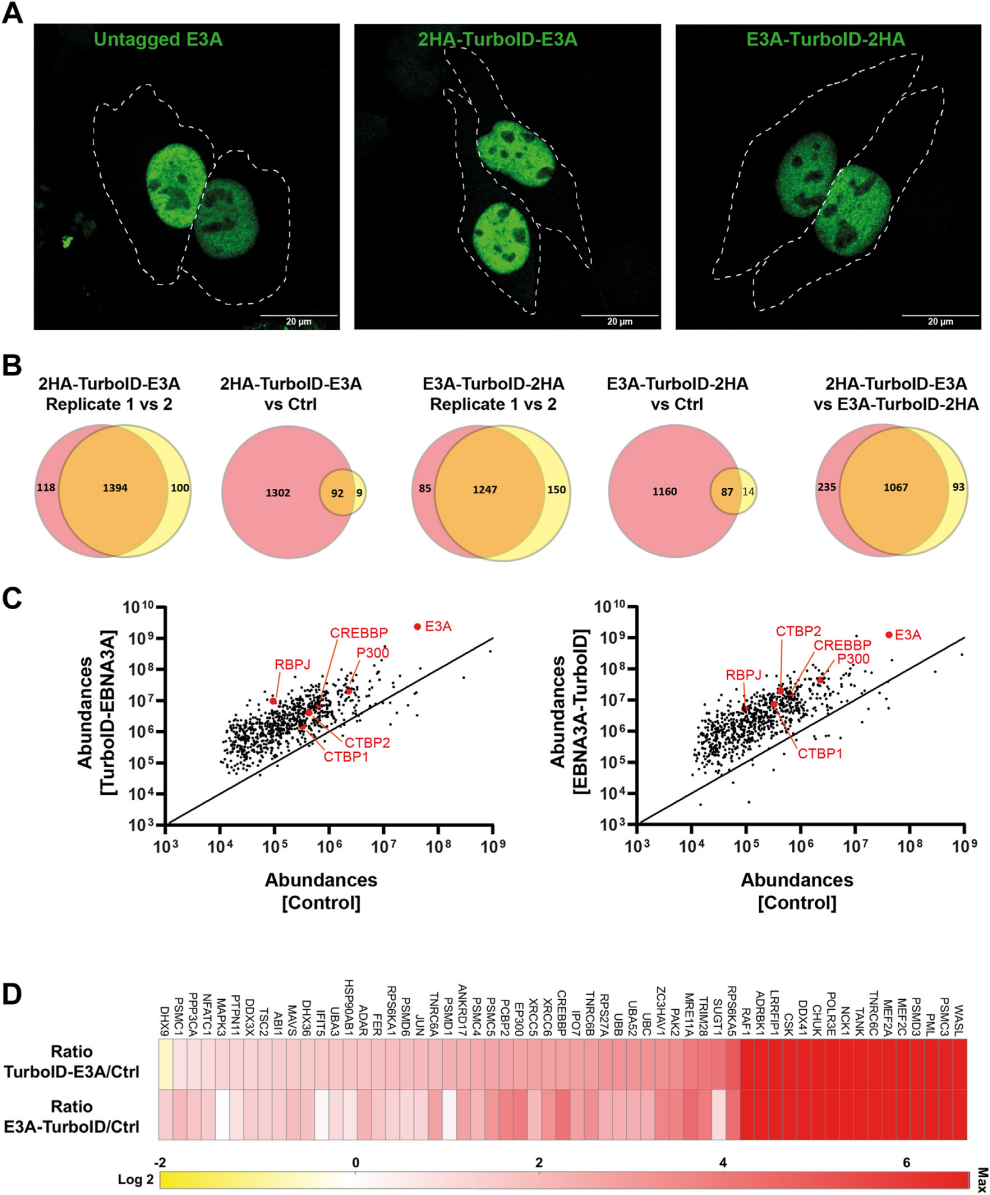
**Proximity ligation assay reveals immune signaling proteins interacting with EBNA3A.***A*, representative immunofluorescence images of HeLa cells expressing untagged EBNA3A, TurboID-EBNA3A, or EBNA3A-TurboID, fixed and stained against EBNA3A (*green*) and DAPI (*blue*) to label nuclei. Scale bar = 20 μm. Cell boundaries are demarcated with *dashed lines*. *B–D*, Cellular interactome of EBNA3A probed using Proximity Biotinylation. *B*, Venn diagram analysis comparing biotinylated proteins extracted from HEK 293T cells expressing TurboID-E3A *versus* E3A-TurboID or untransfected (control) cells following incubation with cell permeable biotin for 120 min. Biotinylated proteins were isolated from cell lysates by NeutrAvidin precipitation and analyzed by mass spectrometry. Reproducibility between n = 2 independent sample sets is shown. *C*, peptide abundances of biotinylated proteins identified from cells expressing TurboID-E3A or E3A-TurboID as compared to control. *D*, Heatmap of EBNA3A-interacting proteins involved in immune signaling according to the Gene Ontology (GO) project. EBNA, EBV-nuclear antigen; HEK, human embryonic kidney.

To gain insight into the molecular mechanism employed by EBNA3A to dampen IFN I induction, we investigated protein-protein interactions of EBNA3A using a proximity-based biotinylation strategy (40). A promiscuous biotin ligase domain was fused to either the N-terminus (TurboID-E3A) or C-terminus (E3A-TurboID) of EBNA3A, neither of which interfered with its nuclear localization (Fig. 3*A*) (41). Biotinylated proteomes from HEK293T cells expressing TurboID-E3A or E3A-TurboID were captured by NeutrAvidin precipitation (Fig. S3). Mass spectrometric analysis (LC/MS/MS) identified 1067 proteins shared between biotinylated isolates from cells expressing TurboID-E3A and E3A-TurboID upon excluding those found within the endogenous biotinylation control from untransfected cells (Fig. 3, *B* and *C*). These identified proteins included two known interaction partners of EBNA3A, *viz.* recombination signal binding protein for immunoglobulin kappa J region (RBP-Jκ) and c-terminal-binding protein (CtBP), validating our chosen strategy (42, 43). We visualized the enrichment of proteins implicated in immune responses in our TurboID-E3A and E3A-TurboID biotinylated precipitates (Fig. 3*D*). Two proteins of interest were histone acetyltransferases CBP and P300, which, like CtBP and RBP-Jκ are regulators of transcription (44, 45, 46). Importantly, neither CBP nor P300 have been studied in the context of EBNA3A.

**Figure 4:**
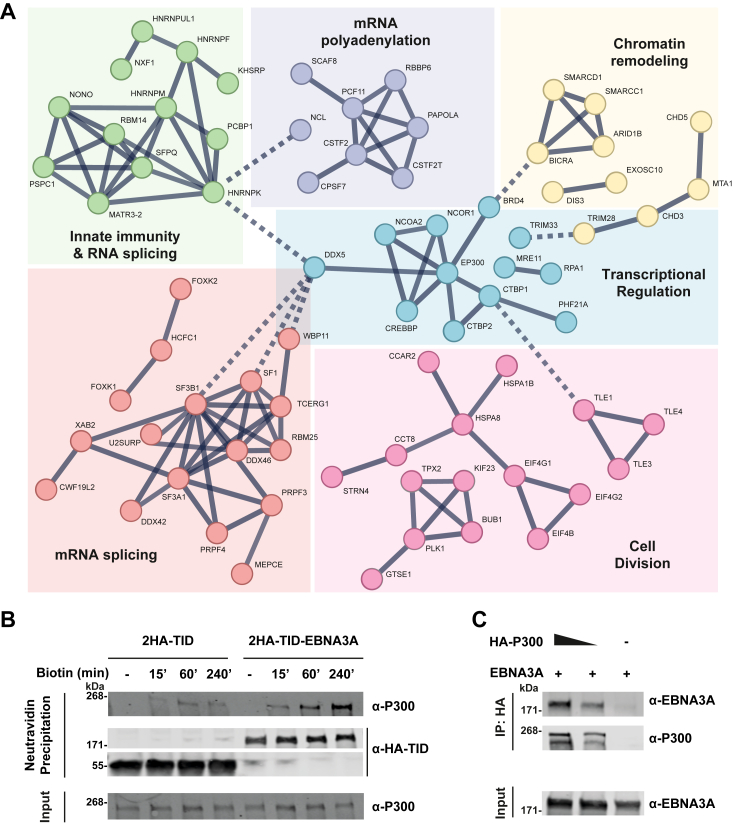
**EBNA3A interacts with the IFN I coactivator P300.***A*, HEK293T cells expressing TurboID-E3A or E3A-TurboID were incubated with biotin for 120 min. Biotinylated proteins were isolated from cell lysates using NeutrAvidin precipitation and analyzed by mass spectrometry. Highly enriched hits were analyzed using the STRING database and clustered into six groups. Interactions between and within groups are indicated with dashed and solid lines approximately. The most common cellular function of each cluster is indicated. Known interactors CtBP1/2 and novel interactors CREBBP and P300 are marked by a *dashed box*. Parameters: peptides >10; Enrichment/ctrl > 10. *B* and *C*, validation of P300 as an interactor of EBNA3A. *B*, HEK293T cells expressing HA-tagged TurboID-EBNA3A or TurboID-vector control were incubated with biotin for the indicated time points. Biotinylated proteins were precipitated using NeutrAvidin beads and analyzed by immunoblotting against P300 and HA. *C*, co-immunoprecipitation of EBNA3A with P300. HEK293T cells expressing EBNA3A and increasing amounts of HA-P300 were lysed and subjected to immunoprecipitation using anti-HA beads, followed by immunoblotting against P300 and EBNA3A antibodies. Positions of marker standards are indicated. CtBP, c-terminal-binding protein; EBNA, EBV-nuclear antigen; HEK, human embryonic kidney; IFN, type I interferon.

Next, we visualized the interactome of EBNA3A and identified six clusters of proteins belonging to various biological pathways, including immune response and gene regulation (Fig. 4*A*). Again, CBP and P300 were found, localized in the center cluster (dashed box) together with CtBP 1 and 2. P300 is a central component of the IFNβ enhanceosome complex required for IFNβ transcription (47). Therefore, we speculated that P300 could be targeted by EBNA3A to inhibit IFN I induction. We confirmed the proximity labeling of P300 by TurboID-E3A by immunoblotting (Fig. 4*B*). Indeed, EBNA3A biotinylated P300 in a time-dependent manner. Since a physical interaction is not necessarily required for proximity biotinylation, we performed co-immunoprecipitation revealing complex formation between EBNA3A and P300 (Fig. 4*C*). Biotinylation of CBP, which is closely related to P300, was confirmed by immunoblotting as well, further strengthening our observation that EBNA3A interacts with the IFNβ enhanceosome (Fig. S4). Altogether, these results provide a rationale for EBNA3A-mediated inhibition of IFN I induction.

### The N-terminus of EBNA3A binds to P300 and mediates inhibition of IFN I induction

**Figure 5:**
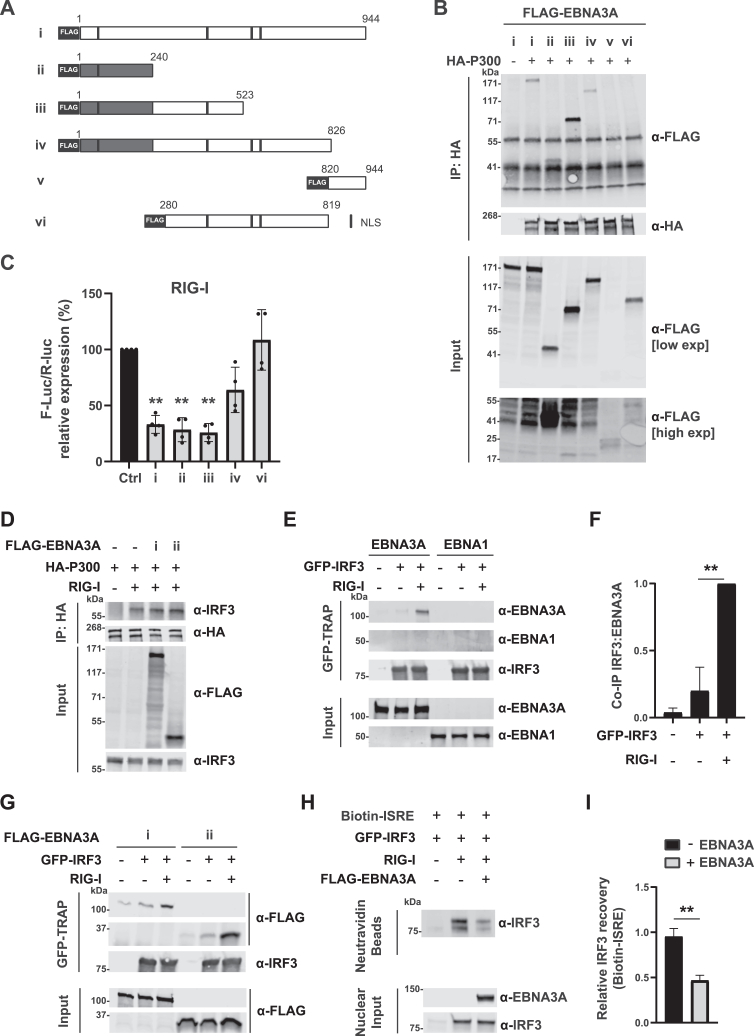
**EBNA3A binds to P300 and IRF3 through its N-terminal domain and inhibits binding of the transcription factor IRF3 to target DNA.***A*, schematic overview of FLAG-tagged EBNA3A truncation mutants. Dark stripes represent nuclear localization signals. *B*, co-immunoprecipitation analysis of P300 and EBNA3A truncation mutants. HEK293T cells were transfected for 24 h with HA-P300 and FLAG-EBNA3A truncation mutants, lysed, and subjected to HA immunoprecipitation. Immunoprecipitates were resolved by SDS-PAGE and analyzed by immunoblotting using the indicated antibodies. *C*, effect of EBNA3A mutants on IFN I induction. HEK293 cells were co-transfected for 24 h with a reporter plasmid expressing the firefly luciferase gene under the control of the IFNβ promoter, a control plasmid expressing the renilla luciferase gene under the control of a constitutive promoter (for normalization purposes), RIG-I, and the indicated EBNA3A truncation mutants. Firefly luciferase activity was measured and normalized to the renilla luciferase activity within each sample. Corrected firefly luciferase activity was then expressed relative to control set at 100%. *D*, EBNA3A does not affect co-immunoprecipitation of P300 and IRF3. HEK293T cells were transfected with HA-P300, RIG-I, and full-length FLAG-EBNA3A or a C-terminal truncation mutant for 24 h, lysed and subjected to HA immunoprecipitation, followed by immunoblotting against IRF3, FLAG and P300. *E*, co-immunoprecipitation of IRF3 and EBNA3A or EBNA1. HEK293T cells were transfected with GFP-IRF3, RIG-I, and EBNA3A or EBNA1 for 24 h. Cells were lysed and subjected to immunoprecipitation using GFP-trap beads, followed by immunoblotting against EBNA3A, EBNA1, and IRF3. *F*, relative co-immunoprecipitation of IRF3 and EBNA3A in the absence or presence of RIG-I. IRF3/RIG-I +/+ is set at 1. *G*, co-immunoprecipitation of IRF3 and EBNA3A. HEK293T cells were transfected with GFP-IRF3, RIG-I, and full-length FLAG-EBNA3A or a C-terminal truncation mutant for 24 h. Cells were lysed and subjected to immunoprecipitation using GFP-trap beads, followed by immunoblotting against FLAG and IRF3. *H* and *I*, DNA binding assay for the effect of EBNA3A on IRF3-DNA interaction. *H*, HEK293T cells were transfected with the indicated combinations of GFP-IRF3, RIG-I, and FLAG-EBNA3A. Cells were lysed and their nuclear extracts were incubated with a biotin-labelled DNA probe encoding an IRF3-binding ISRE sequence, followed by NeutrAvidin precipitation and immunoblotting using the indicated antibodies. *I*, ratios of IRF3 protein levels ± FLAG-EBNA3A before and after NeutrAvidin precipitation. n = 3 independent experiments, significance calculated using unpaired Student's *t* test. Graph shows mean ± SD, ∗∗*p* < 0.01. Positions of marker standards are indicated. EBNA, EBV-nuclear antigen; HEK, human embryonic kidney; IFN, type I interferon; IRF3, interferon regulatory factor 3; ISRE, IFN-stimulated response element; RIG-I, retinoic acid-inducible gene I.

Next, we investigated which domain of EBNA3A interacts with P300. Others have shown that both the N-terminus and C-terminus of EBNA3A engage in protein interactions (with RBP-Jκ and CtBP, respectively) (43, 48). To determine which domain of EBNA3A mediates binding to P300, we generated a series of truncation mutants based on previous studies lacking the N-terminal, C-terminal, or both N- and C-terminal domains (Fig. 5*A*) (41, 49). Similar to the full-length EBNA3A, mutants containing its N-terminal domain co-immunoprecipitated with P300 (i-iv), unlike those lacking this domain (v-vi) (Fig. 5*B*). We further investigated whether EBNA3A truncation mutants capable of binding P300 also retained the ability to inhibit IFN I induction. EBNA3A full-length (i) as well as mutants ii (1–240) and iii (1–523) strongly inhibited RIG-I-mediated IFNβ promoter activation in HEK293 cells, while mutant vi (280–819) did not (Fig. 5*C*). Mutant v (820–944) was not further considered in this panel due to its poor expression (Fig. S5). Unexpectedly, mutant iv (1–826) only moderately inhibited activation. This may be due to differences in its tertiary structure or lower expression compared to other mutants (Fig. S5). Taken together, these data define amino acids 1 to 240 of EBNA3A as the P300 binding domain. The interaction between P300 and this domain may dampen IFN I induction.

### EBNA3A interferes with the binding of IRF3 to DNA

**Figure 6:**
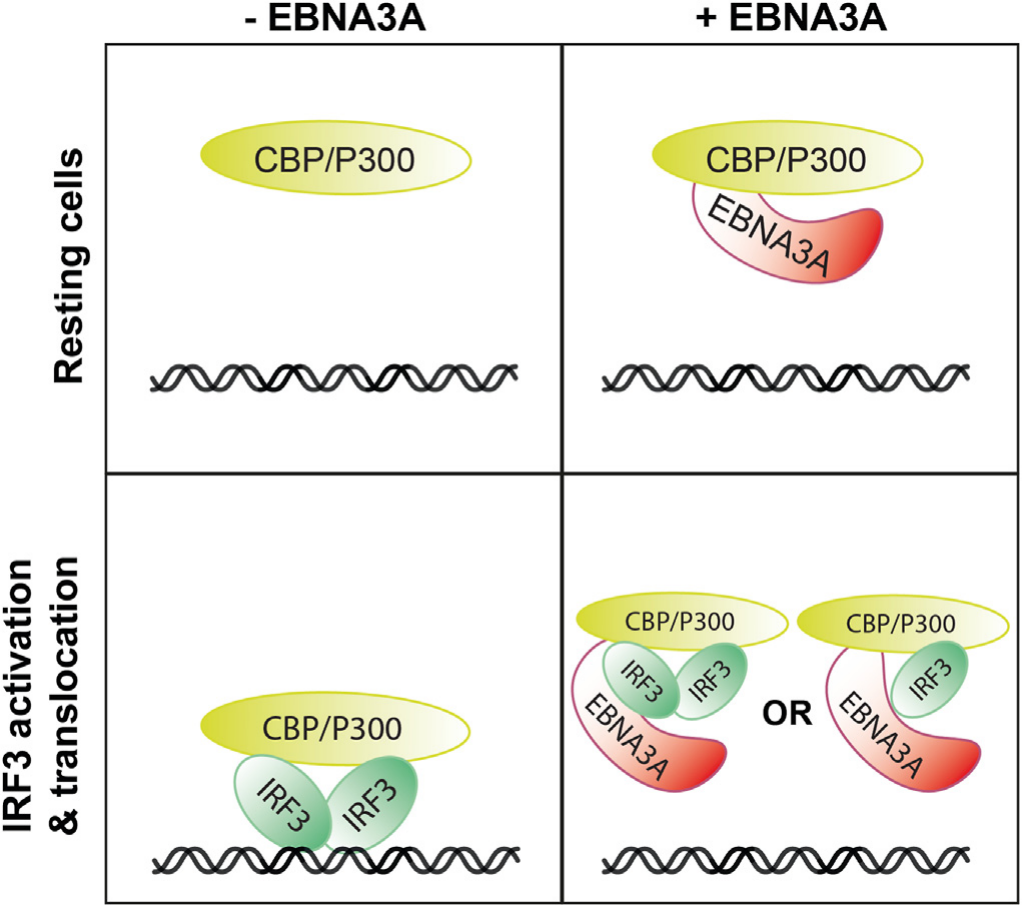
**Model of inhibition of IFN I signaling by EBNA3A in the nucleus.** In resting cells, EBNA3A interacts with the coactivator P300. Upon stimulation, IRF3 translocates to the nucleus, where it binds to the ISRE promoter region together with P300 to induce IFNβ induction. However, EBNA3A binds to activated IRF3 and interrupts the interaction between IRF3 and ISRE DNA, thereby inhibiting IFN I induction. EBNA, EBV-nuclear antigen; IFN, type I interferon; IRF3, interferon regulatory factor 3; ISRE, IFN-stimulated response element.

Having established that EBNA3A is an inhibitor of IFN I induction and a binding partner of P300, we proceeded to delineate the molecular interplay driving inhibition. In uninfected cells, RLR or cGAS activation leads to the nuclear translocation of IRF3 and complex formation with P300. A host protein (Ago2) and viral protein (vIRF-1 of KSHV) have been shown to interfere with the interaction between P300 and IRF3 (50, 51). We hypothesized that EBNA3A could act similarly. To test this, we determined whether EBNA3A affects the recruitment of IRF3 to P300. However, overexpression of EBNA3A did not appear to affect co-immunoprecipitation of IRF3 with P300, implying that another mechanism is likely responsible for EBNA3A-mediated inhibition of IFN I induction (Fig. 5*D*). EBNA3A modulates gene activation through its interaction with transcription factors (52, 53). Hence, we considered whether EBNA3A associates with IRF3 and interferes with the binding of IRF3 to DNA. EBNA3A, but not EBNA1, was robustly recovered in IRF3 co-precipitates from HEK293T cells overexpressing RIG-I (Fig. 5, *E* and *F*). The N-terminal domain of EBNA3A, found to be responsible for the interaction with P300 (Fig. 5*B*), was also sufficient for IRF3 binding (Fig. 5*G*). Having determined that EBNA3A can interact with IRF3, we next investigated whether EBNA3A can block binding of IRF3 to DNA in the nuclear compartment. Nuclear extracts from HEK293T cells overexpressing IRF3 in the absence or presence of RIG-I and EBNA3A were incubated with an IRF3 DNA binding probe based on the IFNβ promoter sequence (Biotin-ISRE), and IRF3-Biotin-ISRE complex formation was detected using NeutrAvidin precipitation followed by immunoblotting. Overexpression of EBNA3A was found to significantly reduce binding of nuclear IRF3 to its cognate DNA motif (Fig. 5, *H* and *I*). Collectively, these findings support a model wherein nuclear EBNA3A can interact with P300 in unstimulated cells. Upon stimulation, EBNA3A also associates with IRF3 and interferes with its binding to the IFN I promoter, without perturbing the interaction between IRF3 and P300 (Fig. 6).

## Discussion

Viruses have acquired many strategies to evade the antiviral immune response, and mapping their underlying molecular mechanisms may provide new tools for improving global health. Here we demonstrate that the EBV-encoded latent nuclear antigen EBNA3A possesses the ability to block IRF3 binding to the IFNβ promoter, thereby attenuating downstream interferon-mediated responses.

It is becoming increasingly clear that the presence of RNA and DNA viruses in host cells is not exclusively surveyed by either RNA or DNA sensors, respectively. For instance, DNA viruses such as adenoviruses and herpesviruses EBV, KSHV, and HSV-1 can be (in)directly sensed by RLRs (12, 54, 55). Similarly, cGAS and the stimulator of interferon genes play a role in restricting the infection of several RNA viruses, such as Dengue, Sendai, vesicular stomatitis, and the West Nile virus (56). Therefore, it comes as no surprise that viruses have evolved strategies to interfere with both routes of cytosolic nucleic acid sensing pathways by targeting their dedicated sensors and signaling intermediates through means of cleavage, manipulation of post-translational modifications, and initiation of degradation (57, 58, 59). Previous studies have shown that EBV expresses lytic (BLRF1) and latent (LMP1) genes as well as several microRNAs (BART3, -6, and -19), all of which hamper RIG-I-dependent IFN I induction (60). Our data add to this emerging paradigm by revealing a potential role of EBNA3A in viral interference with the RIG-I signaling axis. We show that EBNA3A can hamper cytosolic DNA sensing *via* IRF3, a signaling intermediate essential for both RNA and DNA sensing pathways. Interestingly, EBV is not the only virus to manipulate IRF3 function (19), and understanding distinct viral approaches targeting IRF3 may inform future development of antiviral therapeutics.

Despite limited sequence similarity to its family members, EBNA3A exhibits strong structural similarity with EBNA3B and 3C (52, 61). This is in turn functionally reflected by a 50% overlap in genome-wide binding sides between EBNA3A and 3C (53). Furthermore, both EBNA3A and 3C were reported to have oncogenic properties. Immortalization and transformation of cells by EBNA3A and 3C is mediated through binding the transcriptional repressor CtBP (43, 62), which we also found to be a putative interactor of EBNA3A. On the other hand, although EBNA3A and 3B share nearly 90% structural similarity, EBNA3B does not appear to be oncogenic but rather tumor-suppressive (63). The largest sequential overlap between EBNA3 family members is found in the N-terminus, between aa 90 and 313 for EBNA3A (52). Interestingly, these amino acids partially overlap with the segment of EBNA3A we identified as sufficient for P300 binding and inhibition of IFN I induction. Yet, we find that neither EBNA3B nor EBNA3C can appreciably hinder IFN I production, even though EBNA3C is able to bind P300 (64, 65). This suggests that binding to IRF3 determines whether an EBNA3 protein can interfere with the IFN I arm of the antiviral response. In fact, EBNA3C has been shown to interact with other members of the IRF protein family, namely IRF4 and IRF8, which it accomplishes *via* its N-terminal domain (66). These considerations point to an evolutionarily conserved affinity of EBNA3 family members for host IRF proteins, albeit with unique preferences.

The findings presented here establish EBNA3A as an inhibitor of IFN I induction. In resting cells, EBNA3A binds to the IFNβ enhanceosome complex component P300. The interaction with P300 alone seems insufficient to prevent downstream immune activation, which is consistent with the inability of EBNA3C to inhibit IFN I induction, despite its competence in binding P300. Furthermore, EBNA3A was incapable of blocking the interaction between IRF3 and P300. Instead, EBNA3A may utilize P300 to expedite its interaction with IRF3. On this basis, we propose a model wherein EBNA3A renders IRF3 incapable of activating the IFNβ promoter, either by sterically hindering the DNA binding domain of IRF3 or by replacing one of the IRF3 monomers. Targeting IRF3 allows EBNA3A to repress IFN I induction regardless of the nature of upstream stimuli (*i.e.*, DNA or RNA).

An outstanding question remains how EBNA3A fits among other inhibitors of the interferon signaling cascade expressed during latency. EBNA3A is among the first latent antigens to be downregulated upon latency progression (32). Early shutdown may be a necessary step for the establishment of infection, as EBNA3A is potently immunogenic in the context of T-cell activation (52). LMP1 and LMP2 are expressed together with EBNA3A (Latency III), but also remain present during the second stage of latency (Latency II) (67). By contrast, microRNA BART6 is most abundant in the final stage of latency (Latency I) (68). Thus, timing and cooperation between viral genes may be of the essence to achieve a successful infection cycle. Studying the inhibitory effect of EBNA3A on interferon signaling in the context of EBV-infected B-cells would therefore be of high interest. In turn, revealing the mechanisms of immune evasion at play may open new possibilities for the development of antiviral therapies as well as interventions for immune-mediated diseases.

## Experimental procedures

### Cell lines

HEK293T, HeLa and MelJuSo cells were cultured at 37 °C and 5% CO2. HEK293 cells were cultured at 37 °C and 10% CO2. All cells were cultured in Dulbecco’s Modified Eagle’s Medium (DMEM, GIBCO) supplemented with 10% heat-inactivated Fetal Bovine Serum (FBS, BioWest) and 100 U/ml penicillin and 100 μg/ml streptomycin (Gibco). The B-LCL cell line CP364-1 was kindly provided by Prof. Dr B. Kempkes (35). JY and CP364-1 cells were cultured at 37 °C and 5% CO2 in Roswell Park Memorial Institute 1640 medium (RPMI, GIBCO) supplemented with 10% heat-inactivated Fetal Bovine Serum (FBS, BioWest) and 100 U/ml penicillin and 100 μg/ml streptomycin (Gibco). Cells were tested negative for *mycoplasma* at regular intervals.

### Luciferase assay

Cells were seeded in culture plates and allowed to adhere overnight to obtain approx. 70% confluency on the day of transfection. For transfection, 200 ng DNA and 0.5 μl Lipofectamine 2000 (#11668-019, Invitrogen) were mixed in 25 μl Opti-MEM (Gibco) per well of a 96 well plate. Each DNA mixture contained 70 ng p125 IFN-β Firefly-luciferase and 10 ng pRL-TK Renilla luciferase plasmids for the luciferase assay readout. Plasmids encoding the EBV latency associated genes were kindly provided by PD Dr J. Mautner (69). Mixtures were incubated for 15 to 20 min at room temperature (RT) before addition to the cells. 24-40 h post transfection, supernatants were removed and cells were lysed in 100 μl 1× passive lysis buffer (#E1910, Promega). Lysates were stored at −80 °C. 10 μl of lysate was transferred to a white OptiPlate-96 (PerkinElmer) per sample and 50 μl of one-third diluted Firefly dual luciferase assay substrate (#E1910, Promega) was added. Following measurement with Firefly, Renilla dual luciferase assay substrate (#E1910, Promega) was added and measured. Measurements were conducted using a VICTOR X3 Plate Reader (PerkinElmer).

### RNAi-mediated gene silencing

For siRNA transfections, the following custom siRNA oligos used to target EBNA3A were purchased from Dharmacon: sense-siEBNA3A#1 ACACAGGAGCCUCACGAAA and sense-siEBNA3A#2 CGAAGGUGCUACCGGUGAA. JY cells were transfected in a 24w flat-bottom plate with 10 to 40 pmol of each siRNA using the Lipofectamine RNAiMAX Transfection Reagent (Thermofisher) according to manufacturer’s protocol. MelJuSo cells were reverse transfected in a 24w flat-bottom plate with 50 nM of siEBNA3A#2 using the DharmaFECT Transfection Reagent (Horizon) according to manufacturer’s protocol.

### Replication-deficient lentiviruses and transductions

SIN lentiviruses were generated in HEK293T cells transfected with the lentiviral plasmid encoding pSico-EF1a-puroR-T2A-HisEK-EBNA3A and pSico-EF1a-puroR-T2A-eGFP as a control, along with vectors pCMV-VSV-G, pMDLg-RRE, and pRSV-REV to provide the helper functions (70). Supernatants were harvested 72 h post-transfection by centrifugation (5 min, 700*g*) and filtration (0.45 μm). To transduce MelJuSo cells, the supernatant containing lentiviral particles was added along with 4 μg/ml polybrene and plates were spin-inoculated (2000 rpm, 33 °C, 90′) to enhance transduction efficiency. 72 h after transduction, cells were cultured in fresh medium containing puromycin (1 μg/ml).

### IFN I bioassay and flow cytometry

Cells plated in 24w flat-bottom plates were transfected with a 1:1 mix of poly(I:C) (1 μg) and lipofectamine 2000 (#11668-019, Invitrogen). Mixtures were incubated for 15 to 20 min before adding them to the cells to allow for complex formation. For flow cytometry, cells were incubated with 1000 U/ml IFNα (#11100-1 PBL). 24 h after incubation, supernatants were harvested and stored at −20 °C prior to analysis. Cells were collected for flow cytometry analysis. HEK Blue IFN-α/β reporter cells (Invivogen) were incubated with cell-free supernatant for 24 h according to the supplier’s instructions. IFN-α/β levels were determined by measuring the SEAP activity in culture supernatants using a colorimetric assay, measuring OD at 655 nm. All experiments were performed in technical duplicates. To determine expression levels of ISG15, cells were fixed with 2% paraformaldehyde (Sigma) in PBS, permeabilized in saponin buffer (PBS, 2% fetal calf serum, 0.5% saponin), stained with anti-ISG15-PE (IC8044P, R&D systems) and washed 2× before being taken up in PBS + 0.5% BSA + 0.02% sodium azide. Fluorescence was measured using a LSR II flow cytometer (BD Biosciences). Data was analyzed using FlowJo software V10.7.1 (Treestar).

### RT-qPCR

Total RNA was extracted using TRIzol (Invitrogen) and reverse-transcribed using SuperScript IV VILO Master Mix (Invitrogen), both according to the manufacturer’s protocol. cDNA was diluted in nuclease-free water and gene expression was measured in technical triplicates using 2× SYBR Green Master Mix (Bio-Rad) on a CFX Connect Real-Time PCR Detection System (Bio-Rad). Gene-specific primers are listed in Table S1.

### Confocal microscopy

For fluorescence confocal imaging of fixed samples, HeLa cells were seeded in 8-well Lab-Tek II chamber slides and allowed to adhere overnight (Thermo Scientific). Cells were transfected with 400 ng plasmid DNA encoding untagged EBNA3A, TurboID-EBNA3A, or EBNA3A-TurboID and 2 μl PEI (1 μg/μl, #23966, Polysciences) for 24 h, fixed with 4% paraformaldehyde (Sigma) in PBS for 10 min, and permeabilized using 0.25% Triton X-100 (MP Biomedicals) in PBS for 10 min. Samples were immunostained using rat α-EBNA3A primary antibody (35) (Helmholtz Zentrum München) for 30 min at RT, washed 3× in PBST (PBS + 0.05% Tween20 [Merck]) followed by the secondary antibody donkey-anti-rat 488 (#A-21208, Invitrogen) for 20 min. Dilutions were prepared in TNB (TBS, 0.5% blocking reagent [#11096176001, Roche], 0.02% Thimerosal [Sigma]). Samples were washed 3× with PBST and mounted on glass slides with ProLong Gold antifade Mounting medium containing DAPI (#P36931, Invitrogen). Samples were imaged on a Leica SP8 confocal microscope equipped with appropriate solid-state lasers, HCX PL 63 times magnification oil emersion objective and HyD detectors (Leica Microsystems). Data were collected using a digital zoom of 2 in 1024 × 1024 scanning format with line averaging. Post-collection image processing was performed using Fiji software v. 1.53q.

### Proximity-based biotinylation

To profile the protein interactome of EBNA3A, HEK293T cells were seeded in 6 cm dishes and transfected with TurboID-EBNA3A or EBNA3A-TurboID as indicated. 24 h post transfection, supernatants were supplemented with biotin (50 μM final [#B4639, Sigma]) for 60 to 240 min. After incubation, cells were washed two times with PBS and lysed in 1 ml Igepal lysis buffer (150 mM NaCl, 50 mM Tris-HCL [pH 8.0], 5 mM MgCl_2_, 0.8% Igepal CA-630 [#18896, Sigma]) containing EDTA free cOmplete Mini protease inhibitor cocktail (Roche). Lysates obtained following centrifugation (15 min at 12,000*g*) were incubated with NeutrAvidin coated beads (#29200, ThermoFisher) rotating overnight at 4 °C. Beads were then washed 4× in lysis buffer. Complete removal of buffer after the final wash was accomplished by aspiration with a needle syringe. Precipitates were denatured using Laemmli sample buffer containing 5% β-mercaptoethanol, followed by 5 min incubation at 95 °C. Proteins were separated on 4 to 15% precast gradient gels (#4561086, Bio-Rad) followed by immunoblotting or silver staining performed according to the manufacturer’s protocol (SilverQuest, Invitrogen) as indicated.

### Mass spectrometry

Sample workup was done as previously described (71). For MS analysis, gel bands were reduced with 10 mM dithiothreitol, alkylated with 50 mM iodoacetamide. In-gel trypsin digestion was performed using a Proteineer DP digestion robot (Bruker). Tryptic peptides were extracted from the gel slices using 50/50/0.1 water/acetonitrile/formic acid (v/v/v). Samples were lyophilized, dissolved in 0.1% formic acid and subsequently analyzed by online C18 nanoHPLC MS/MS with a system consisting of an Easy nLC 1000 gradient HPLC system (Thermo, Bremen, Germany), and a LUMOS mass spectrometer (Thermo). Samples were injected onto a homemade precolumn (100 μm × 15 mm; Reprosil-Pur C18-AQ 3 μm, Dr Maisch, Ammerbuch, Germany) and eluted *via* a homemade analytical nano-HPLC column (30 cm × 50 μm; Reprosil-Pur C18-AQ 3 μm). The gradient was run from 10% to 40% solvent B (20/80/0.1 water/acetonitrile/formic acid (FA) v/v) in 30 min. The nano-HPLC column was drawn to a tip of ∼5 μm and acted as the electrospray needle of the MS source. The LUMOS mass spectrometer was operated in data-dependent MS/MS mode for a cycle time of 3 s, with a HCD collision energy at 32 V and recording of the MS2 spectrum in the orbitrap. In the master scan (MS1) the resolution was 120,000, the scan range 400 to 1500, at an AGC target of 400,000 @maximum fill time of 50 ms. Dynamic exclusion after n = 1 with exclusion duration of 10 s. Charge states 2 to 5 were included. For MS2 precursors were isolated with the quadrupole with an isolation width of 1.2 Da. First mass was set to 110 Da. The MS2 scan resolution was 30,000 with an AGC target of 50,000 @maximum fill time of 60 ms.

In a post-analysis process, raw data were first converted to peak lists using Proteome Discoverer version 2.4 (Thermo Electron), and then submitted to the Uniprot *Homo sapiens* minimal database (20,205 entries), using Mascot v. 2.2.04 (www.matrixscience.com) for protein identification. Mascot searches were done with 10 ppm and 0.02 Da deviation for precursor and fragment mass, respectively and the enzyme trypsin was specified. Up to two missed cleavages were allowed. Oxidation on Met and acetylation on N-term were set as a variable modification; carbamidomethyl on Cys was set as a fixed modification. Peptides with an FDR<1% in were accepted.

### Co-immunoprecipitation and immunoblotting

HEK293T cells were seeded in 6 cm dishes and transfected as indicated. 24 h post-transfection, cells were lysed in 1 ml Igepal lysis buffer (see above). Supernatants obtained following centrifugation (15 min at 12,000*g*) were incubated with 1 μg anti-HA (#11867423001, Roche) together with 30 μl ProtG beads (GE Healthcare) or 1 μl GFP-Trap_A beads (Chromotek) for 1 h at 4 °C. Beads were then washed 4× in lysis buffer. Complete removal of buffer after the final wash was accomplished by aspiration with a needle syringe. Lysates were denatured using Laemmli sample buffer containing 5% β-mercaptoethanol, followed by 5-min incubation at 95 °C. Proteins were separated on Bio-Rad premade gels (4–20% gradient) and transferred to Nitrocellulose membranes (#1704158, Bio-Rad) using the Trans-blot Turbo system (Bio-Rad). Membranes were blocked in 5(w/v)% cow milk (ELK, Campina) in 1× PBS, incubated with primary antibodies against P300 (#sc-48343, Santa Cruz), HA (#11867423001, Roche), GAPDH (CST), Vinculin (Sigma), EBNA3A (Helmholtz Zentrum München), EBNA1 (OT1x), CBP (sc-7300), FLAG (#F3165, Sigma), His (#34660 Qiagen), GFP (#A-11122, Invitrogen) or IRF3 (#11904, Cell signaling) diluted in Immuno Booster 1 (#T7111A-1, Takara) overnight at 4 °C. Membranes were washed 3 × 5 min in 0.05% PBST, incubated with the IRDye 680/800 secondary antibodies (Li-COR) diluted in Immuno Booster 2 (#T7111A-2, Takara) for 30 min at RT, washed 3 × 5 min in 0.05% PBST, and 1× in PBS. Antibody signals were detected using the Odyssey CLx imager (Li-COR). Images were analyzed with Image Studio Lite (Li-COR).

### DNA binding assay

Sequences of triple repeats of IRF3-recognized and bound ISRE were taken from Wang *et al.* (2017) (50). DNA probes labelled with biotin were custom synthesized by Sigma-Aldrich. Single strands of DNA were thermally annealed to form dsDNA prior to experimentation. HEK293T cells were seeded in 6 cm dishes and transfected as indicated. 24 h post transfection, cells were lysed in 1 ml hypotonic lysis buffer (25 mM HEPES [pH 7.6], 5 mM MgCl_2_, 25 mM KCl, 0.05 mM EDTA, 10(v/v)% glycerol, 0.1(v/v)% NP-40 alternative [Merck]) supplemented with EDTA free cOmplete Mini protease inhibitor cocktail (Roche) and 10 mM NaF. Pellets containing the nuclear fraction were obtained following centrifugation (15 min at 12,000*g*), washed in PBS and resuspended in 60 μl Complete Lysis Buffer (Buffer AM1, DTT, Protease Inhibitor Cocktail, [ActiveMotif]). Lysates were mixed with biotinylated DNA probes and incubated for 1 h at 4 °C, followed by 1 h incubation at 4 °C upon the addition of NeutrAvidin coated beads (#29200, ThermoFisher). Beads were then washed 4× in lysis buffer, and complete removal of buffer after the final wash was accomplished by aspiration with a needle syringe. Lysates were denatured using Laemmli sample buffer containing 5% β-mercaptoethanol, followed by 5 min incubation at 95 °C. Proteins were separated on Bio-Rad premade gels (4–20% gradient) for Western blotting.

### Statistics

All statistical analysis was performed using GraphPad Prism Version 9.3.1 (GraphPad Software). Statistical evaluations used throughout the study report on Student’s *t* test or one-way ANOVA test, as described in the corresponding figure legends, ∗*p* < 0.05, ∗∗*p* < 0.01, ∗∗∗*p* < 0.001, ∗∗∗∗*p* < 0.0001, ns: not significant. All reported error bars reflect the mean ± SD

## Data availability

The mass spectrometry proteomics data have been deposited to the ProteomeXchange Consortium *via* the PRIDE (72) partner repository with the dataset identifier PXD038572.

## Supporting information

This article contains supporting information.

## Conflict of interest

The authors declare that they have no conflicts of interest with the contents of this article.

## Figures and Tables

**Supplemental Figure S1:**