# Supplementary material for: Epstein-Barr virus nuclear antigen EBNA3A modulates IRF3-dependent IFNβ expression
Source: J Biol Chem. 2024 Aug 8;300(9):107645. doi: 10.1016/j.jbc.2024.107645 (PMC11403517; doi:10.1016/j.jbc.2024.107645)
Supplement: Supplementary file 1 — Supplemental Information [file mmc1.docx]

**Supplementary Information**

**Title: Epstein-Barr virus nuclear antigen EBNA3A modulates IRF3-dependent IFNβ expression**

**Running title: EBNA3A modulates IRF3-dependent IFNβ expression**

Sanne L. Landman^1, 2^, Maaike E. Ressing^1^, Anna M. Gram^1^, Rayman T.N. Tjokrodirijo^3^, Peter A. van Veelen^3^, Jacques Neefjes^1, 2^, Rob C. Hoeben^1^, Annemarthe G. van der Veen^4^, Ilana Berlin^1, 2^*

^1^ Department of Cell and Chemical Biology, Leiden University Medical Center (LUMC), Leiden, the Netherlands

^2^ Oncode Institute, Leiden University Medical Center (LUMC), Leiden, the Netherlands

^3^ Center for Proteomics & Metabolomics, LUMC, Leiden, the Netherlands

^4^ Department of Immunology, LUMC, Leiden, the Netherlands

* Corresponding author: Dr. Ilana Berlin, Department of Cell and Chemical Biology, Leiden University Medical Center, Leiden, the Netherlands. E-mail: [i.berlin@lumc.nl](mailto:i.berlin@lumc.nl)

**Supplementary Table 1. Primer sequences used for RT-qPCR.**

| Target | Forward sequence (5’-3’) | Reverse sequence (5’-3’) |
| --- | --- | --- |
| GAPDH | GCAAATTTCCATGGCACCGT | GCCCCACTTGATTTTGGAGG |
| IFNb | TCTGGCACAACAGGTAGTAGGC | GAGAAGCACAACAGGAGAGCAA |
| ISG54 | ATGTGCAACCTACTGGCCTAT | TGAGAGTCGGCCCATGTGATA |

**Supplementary Legends**

**Figure S1. EBNA3A minimally impacts NFκB pathway activation (related to Figure 1).**

**A.** HEK293 cells were co-transfected with a reporter plasmid expressing the firefly luciferase gene under the control of the IFNβ promoter, a control plasmid expressing the renilla luciferase gene under the control of a constitutive promoter (for normalization purposes), RIG-I, and EBNA3A or EBNA-LP. Firefly luciferase activity was normalized to renilla luciferase activity and displayed relative to control cells as in Fig. 1A.

**B.** Plot of renilla luciferase activity only using data in (A) reported relative to control.

**C.** Schematic representation of TNFα and Toll-like receptor (TLR) signaling pathways leading to P50 and P65 (NFκB) dependent activation of pro-inflammatory cytokines.

**D-G.** Overexpression screen for the effects of EBNA3A on the NFκB response. HEK293 cells were co-transfected for 24h with an NFκB-responsive luciferase reporter plasmid, a constitutively expressed reporter plasmid encoding the renilla luciferase, and EBNA3A or A20. Pathway activation was induced by co-transfection of **E.** MYD88, **F.** TRAF6 or **G.** by stimulation with TNFα. n=3 independent experiments, significance calculated using one-way ANOVA. All graphs show mean ± SD. * p <0.05; *** p <0.001;.

**Figure S2. The B-LCL cell line JY responds to poly(I:C) stimulation, but not to RNA interference. (related to Figure 2)**

**A.** Response of B-LCLs to IFNα stimulation. The B-LCL cell lines CP364-1 and JY were stimulated with IFNα for 24h versus unstimulated. Intracellular ISG15 protein expression was measured by flow cytometry.

**B.** Response of B-LCLs to poly(I:C) stimulation. The B-LCL cell lines CP364-1 and JY were lipofectamine transfected with Poly(I:C) for 24h versus treated with lipofectamine alone (Lipo). Intracellular ISG15 protein expression was measured by flow cytometry.

**C.** Effect of siRNA transfection on the B-LCL cell line JY. Cells were transfected with increasing amounts of siCtrl, siE3A1, siE3A2 or siGAPDH for 48h and 72h. Cell lysates were analyzed by immunoblotting against EBNA3A and GAPDH. Positions of marker standards are indicated.

**D** siRNA transfection of MelJuSo cells. Cells were transfected with siCtrl or siE3A2 for 72h. Cell lysates were analyzed by immunoblotting against EBNA3A and Vinculin. Positions of marker standards are indicated.

**Figure S3. Proximity biotinylation by EBNA3A (related to Figure 3B).**

HEK293T cells were transfected with a control plasmid, TurboID-EBNA3A or EBNA3A-TurboID, and left unstimulated or incubated with biotin for 120 min. Biotinylated proteins were isolated from lysates using NeutrAvidin agarose beads prior to SDS-PAGE analysis and silver staining. Arrows indicate protein bands of interest. Positions of marker standards are indicated.

**Figure S4. The P300 coactivator family member CBP is biotinylated by EBNA3A (related to Figure 4C).**

HEK293T cells expressing HA-tagged TurboID-EBNA3A were incubated with biotin for 120 min. Biotinylated proteins were isolated by NeutrAvidin precipitation and analyzed by immunoblotting against CBP and EBNA3A. Dashed lines indicate the splicing of lanes from an individual blot. Positions of marker standards are indicated.

**Figure S5. FLAG-EBNA3A mutant V (820-944) is poorly expressed (related to Figure 5C).**

HEK293 cells were transfected for 24h with a reporter plasmid expressing the firefly luciferase gene under the control of the IFNβ promoter, a control plasmid expressing the renilla luciferase gene under the control of a constitutive promoter (for normalization purposes), RIG-I, and the indicated EBNA3A truncation mutants. Cell lysates were analyzed by immunoblotting against FLAG. Dashed lines indicate the splicing of lanes from an individual blot. Positions of marker standards are indicated.
