# Supplementary figures and images for: Epstein-Barr virus nuclear antigen EBNA3A modulates IRF3-dependent IFNβ expression
Source: J Biol Chem. 2024 Aug 8;300(9):107645. doi: 10.1016/j.jbc.2024.107645 (PMC11403517; doi:10.1016/j.jbc.2024.107645)

**Figure S2**

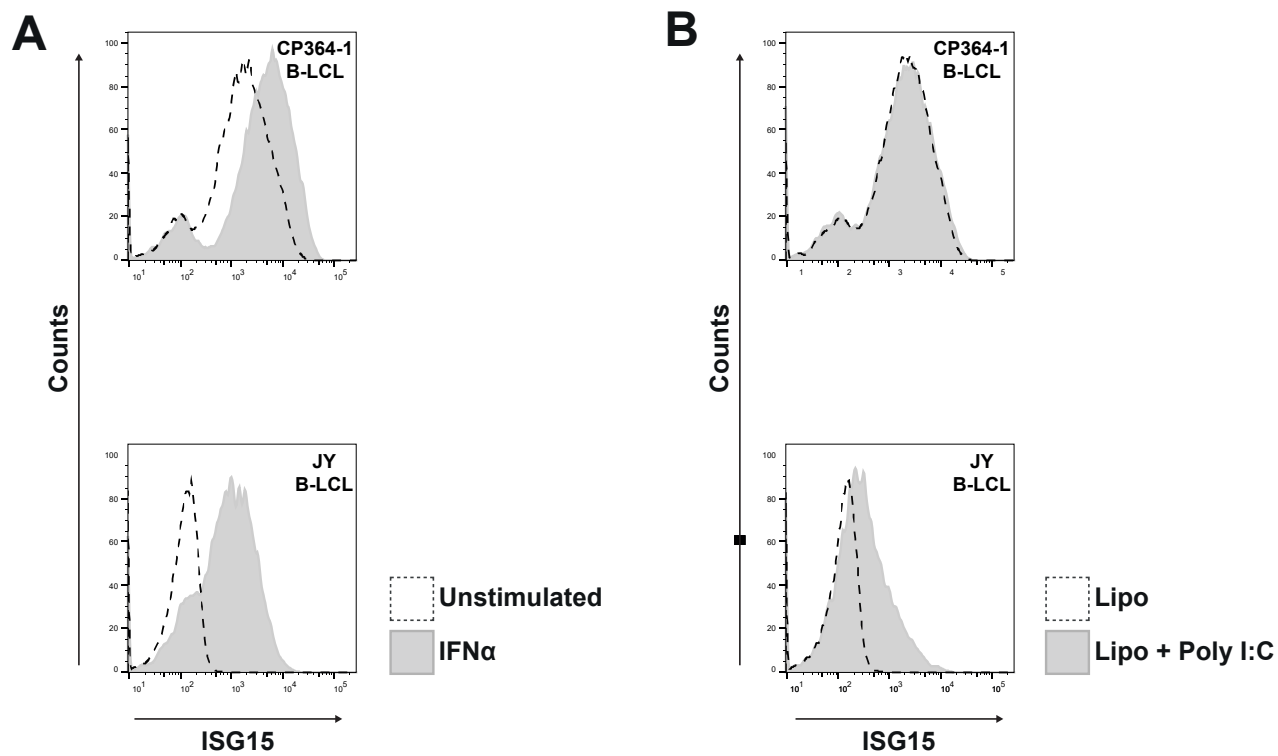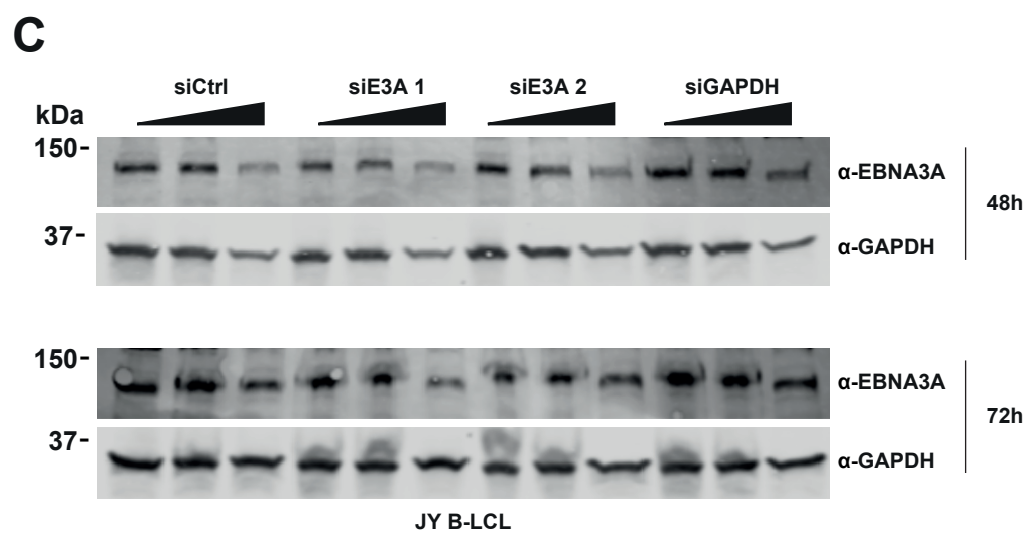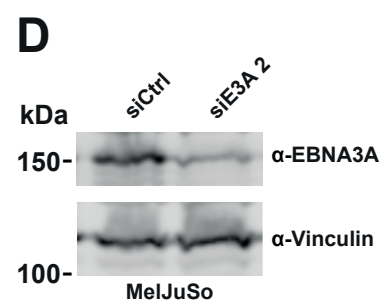

Supplement: Supplemental Figure S2 [file mmc2.pdf]

Figure S3

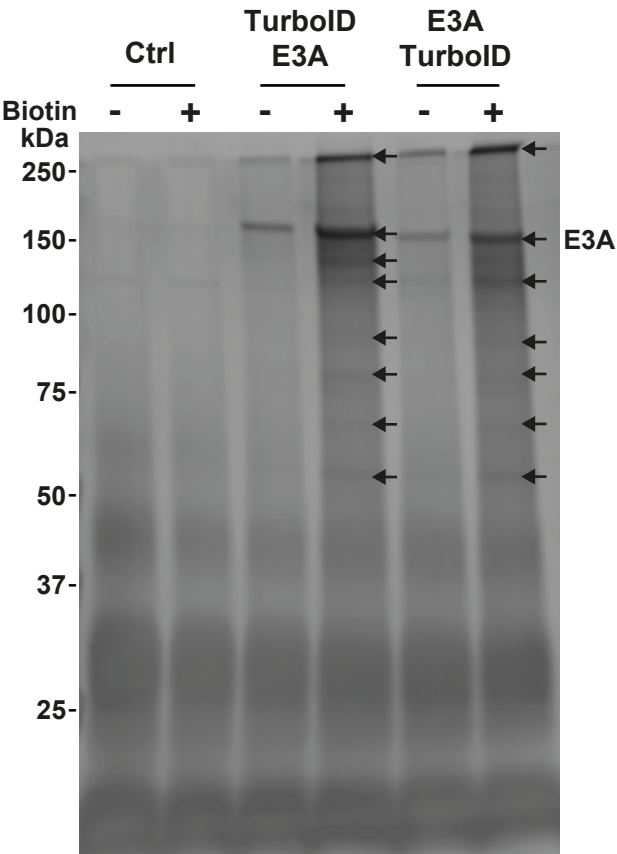

Supplement: Supplemental Figure S3 [file mmc3.pdf]

Figure S4

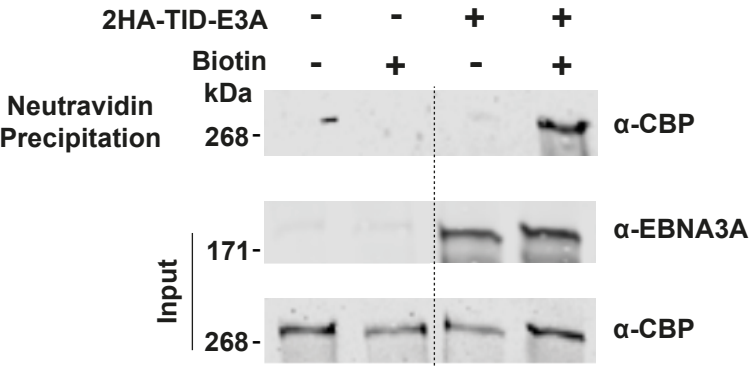

Supplement: Supplemental Figure S4 [file mmc4.pdf]

Figure S5

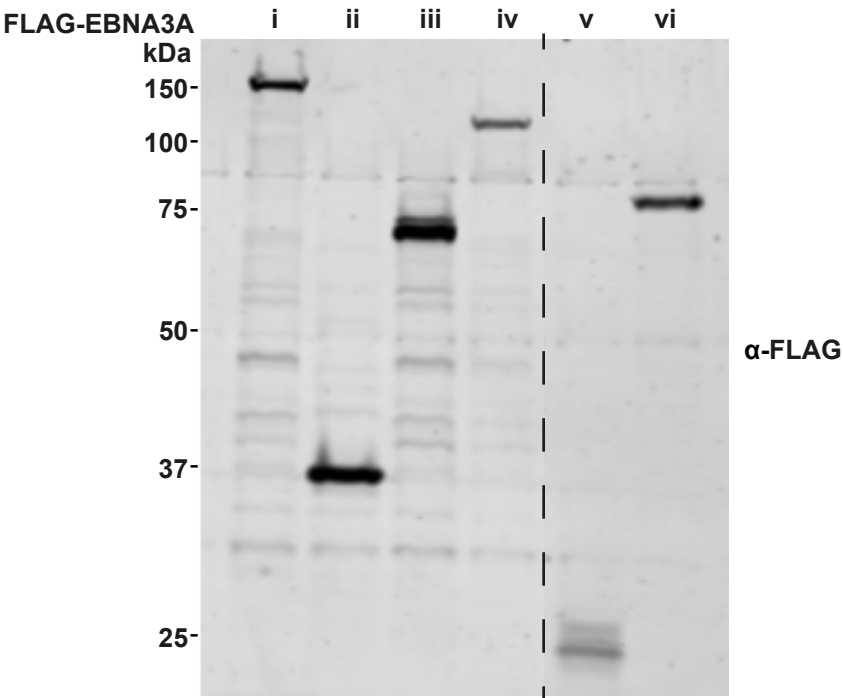

Supplement: Supplemental Figure S5 [file mmc5.pdf]
